# Factors Influencing Trust and Trustworthiness: Cosmetic Injectable Patient Experience Exploratory Study (CIPEES)—Part 3

**DOI:** 10.1093/asjof/ojac082

**Published:** 2022-11-07

**Authors:** Cara B McDonald, Izolda Heydenrych

**Affiliations:** Dermatologist, St Vincent's Hospital Melbourne, Victoria, Australia; Dermatologist in private practice, Cape Town, South Africa

## Abstract

**Background:**

Despite the widely recognized importance of trust in professional relationships, it remains elusive, complex, multidimensional, and difficult to quantify due to the lack of validated tools.

**Objectives:**

The authors sought to explore both the important factors for building trust with cosmetic injectable patients and strategies for improving levels of trustworthiness in aesthetic practitioners.

**Methods:**

In order to explore factors in building trust with cosmetic injectable practitioners, a global study was conducted via an online survey. The Cosmetic Injectable Patient Experience Exploratory Study (CIPEES) survey assessed the relative importance of qualifications and training; reviewing of previous work (before and after photos); reputation; connection; time spent; online presence; and personal appearance of the practitioner in developing trust in a cosmetic practitioner.

**Results:**

The CIPEES survey collected 1488 responses across 75 countries, with 66% of participants completing all 15 questions. The respondents were 95.6% female and 4.4% male, with ages ranging from 18 to >65 years old (median 33 years old). The number 1 ranked reason for being able to trust a cosmetic injector was qualifications and training, followed by the reputation of the practitioner, and time spent by the practitioner with the survey respondent. Practitioner online presence was considered the least important factor in helping develop trust.

**Conclusions:**

The CIPEES survey results support patient trust being built through credibility, reliability, and intimacy, with low levels of practitioner self-orientation. The trustworthiness equation provides a framework for identifying practitioner weaknesses in patient relationships and actionable methods of improving trustworthiness.

Trust is a crucial component in the relationship between the service provider and receiver. In the field of medicine, where patients are often physically and emotionally vulnerable, there is an inherent power imbalance which makes trust essential. Willingness to trust in the care provider is essential in achieving wellness and healing.^1^ The development of interpersonal trust thus remains essential to patient compliance, patient outcomes, and improving patient satisfaction.^2,3^ Clinician interpersonal skills and a trust-based patient–clinician relationship are as important to patients as technical skills and knowledge.^4^ Importantly, trust bilaterally requires the trustworthiness of the care provider and a willingness to trust from the patient.

The field of aesthetic medicine is unique in encompassing both a medical discipline and elective relationship, allowing invasive procedures on generally healthy individuals. Additionally, aesthetic medicine embodies a rapidly growing, competitive, often poorly regulated commercial market which may fail to protect the consumer. For this reason, trust cannot be assumed. In order to develop a lasting, mutually beneficial therapeutic relationship, trust needs to be consciously developed and nurtured by the practitioner. In cosmetic injectable patients, trust in the practitioner correlates highly with patient satisfaction.^5^

Despite the widely recognized importance of trust, it remains elusive, complex, multidimensional, and difficult to quantify due to a lack of validated tools. Therefore, we sought to explore both the important factors for building trust with cosmetic injectable patients and strategies for improving levels of trustworthiness in aesthetic practitioners. Although trust can be clearly defined as a “firm belief in the reliability, truth, or ability of someone or something,”^6^ it is impacted by many variables. The pillars of trust have been examined extensively in business and neuroscience literature.^7^ Frei and Morriss have eloquently defined a trust triangle comprising authenticity, logic, and empathy.^8^ Additionally, clarity, compassion, character, competency, commitment, connection, contribution, and consistency are frequently cited as essential characteristics.^9^ The trustworthiness equation, as cited in The Trusted Advisor^10^ (Figure 1), offers a practical way to define the components of trust, enabling several avenues of increasing trustworthiness within a patient–clinician relationship. This equation defines trust as the sum of credibility, reliability, and intimacy divided by a denominator of self-orientation.

**Figure 1.**
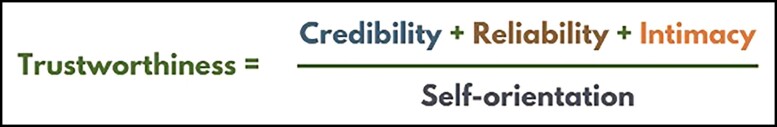
The trustworthiness equation. Reproduced with permission from The Trusted Advisor, Maister, Green and Galford; The Free Press, 2020.^10^

Owing to the patient profile and consumerist demands inherent to aesthetic medicine, optimal patient satisfaction cannot be achieved through technical mastery alone. Building trust also requires both sincerity and technique. The authors consequently endeavored to define guidelines for enabling systematic analysis of patient interactions in order to facilitate the development of interpersonal trust based on the themes of the trustworthiness equation; we aimed to explore the key factors in building trust and rapport in cosmetic injectable patients.

## METHODS

The Cosmetic Injectables Patient Experience—Exploratory Study (CIPEES) was developed to explore trust and other aspects of the patient–practitioner relationship (Appendix A). The survey, open to any person who had previously undergone cosmetic injectable treatments, was anonymous and completed online via snowball recruitment. The snowball approach uses a collaborative network to acquire data from a large study population.^11^ The survey was in the English language but open to participants globally. The survey was hosted on SurveyMonkey (San Mateo, CA) and was open for 10 months, from September 2020 to June 2021. The study was approved by St Vincent's Hospital Melbourne Human Research Ethics Committee. There were no incentives offered, nor any paid advertisement. Written consent was provided, by which the subjects agreed to the use and analysis of their data.

Respondents were asked to rank, in order of personal importance, 7 potential factors that would allow them to trust their cosmetic injectable practitioner. The order in which the reasons appeared was randomized for each respondent. The factors included in the survey were chosen based on the author's personal experience and exploring the topic with cosmetic injectable patients in the clinic setting.

The question was presented as follows: in order for you to trust a practitioner to perform your cosmetic injectable treatments, how would you rank each of the following based on their importance to you, where 1 is the most important and 7 is the least important?

- Qualifications and training of practitioner (degree, doctor, specialist, educator, trainer, key opinion leader)
- Personal appearance and cosmetic ideals/preferences of the practitioner
- Time spent and level of explanation during consultation and procedure
- Feeling comfortable, connected, and at ease with my practitioner
- Practitioner's active online presence and social media following
- Seeing before and after photos of the practitioner's previous cosmetic work
- Reputation or recommendation of the practitioner

In order to determine the factors most important for rapport, respondents also were asked to rate, as not important, somewhat important, quite important, or very important, 7 potential factors that may allow them to feel relaxed and comfortable with their cosmetic practitioner. The order in which the reasons appeared was randomized for each respondent.

The question was presented as follows: in order to relax and feel comfortable with your cosmetic injectable practitioner, how important are the following?

- Practitioner spends adequate time during consultation and procedure
- Practitioner engages in personal/social (nonmedical) conversation
- Practitioner listens and acts on my opinion regarding treatment options and planning
- Practitioner is interested in my background and reasons for undergoing cosmetic treatment
- Practitioner is empathetic and genuinely concerned about how I feel during treatment
- Practitioner has similar interests and values as me
- Practitioner's appearance and behavior suit my personality

## RESULTS

Of the 1430 participants in the CIPEES survey, 95.6% identified as female and 4.4% male, with ages ranging from 18 to >65 years (average 33 years old). Sixty-six percent completed all 15 questions in the survey. The respondents comprised residents from 74 countries, with 59.0% living in Australia, 10.0% in the United States, 6.2% in the United Kingdom, and small numbers across 71 other countries (Appendix B). The numbers were insufficient to analyze differences between countries and cultures.

### Factors in Building Trust

The respondents ranked the importance of 7 factors allowing them to trust a practitioner to perform cosmetic injectable treatments. Nine hundred and eighty-one participants completed this question. In order from most important to least important ranked were:

- Qualifications and training of practitioner (relative ranking 5.85)
- Reputation or recommendation of the practitioner (4.82)
- Time spent and level of explanation during consultation and procedure (4.52)
- Feeling comfortable, connected, and at ease with my practitioner (4.34)
- Seeing before and after photos of the practitioner's previous cosmetic work (3.93)
- Personal appearance and cosmetic ideals/preferences of the practitioner (2.87)
- Practitioner's active online presence and social media following (1.69)

**Figure 2.**
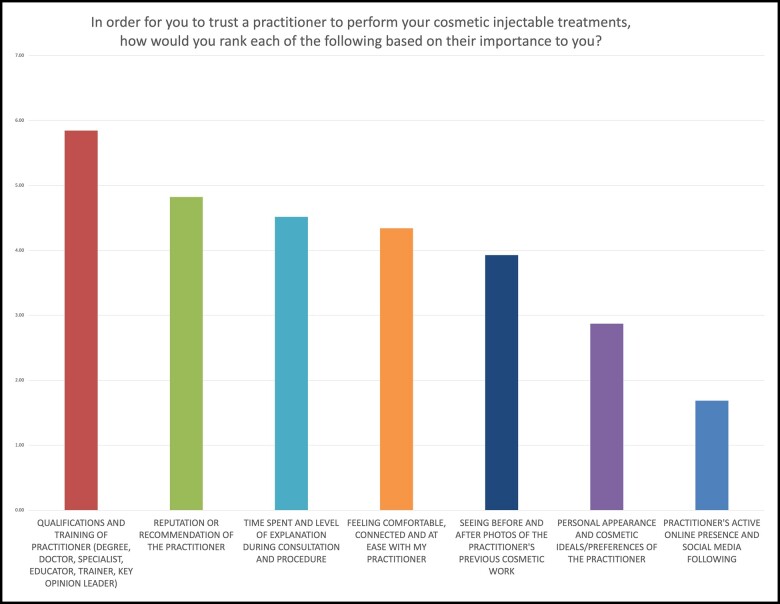
Chart showing relative weighting of factors allowing trust in cosmetic injectable practitioners.

Figure 2 shows the relative importance of each factor, with only minor differences between age groups presented in Table 1.

**Table 1.** Ranking of Factors by Importance in Order to Trust a Practitioner to Perform Your Cosmetic Injectable Treatments, by Age Group (N = 981)

Age group	Overall (n = 981)		18-24 (n = 66)		25-34 (n = 329)		35-44 (n = 277)		45-54 (n = 216)		55-64 (n = 77)		65+ (n = 16)	
	Relative weighting	Ranking	Relative weighting	Ranking	Relative weighting	Ranking	Relative weighting	Ranking	Relative weighting	Ranking	Relative weighting	Ranking	Relative weighting	Ranking
Qualifications and training of practitioner (degree, doctor, specialist, educator, trainer, key opinion leader)	5.85	1	5.82	1	5.64	1	5.62	1	5.8	1	5.94	1	6.25	1
Reputation or recommendation of the practitioner	4.82	2	4.55	3	4.71	2	4.95	2	4.96	2	4.82	2	4.94	2
Time spent and level of explanation during consultation and procedure	4.52	3	4.5	4	4.45	3	4.47	3	4.47	3	4.58	4	4.63	4
Feeling comfortable, connected, and at ease with my practitioner	4.34	4	3.79	5	4.22	5	4.16	4	4.3	4	4.69	3	4.88	3
Seeing before and after photos of the practitioner's previous cosmetic work	3.93	5	4.88	2	4.32	4	3.86	5	3.8	5	3.27	5	3.44	5
Personal appearance and cosmetic ideals/ Preferences of the practitioner	2.87	6	2.56	6	2.76	6	3.14	6	3.08	6	2.94	6	2.75	6
Practitioner's active online presence and social media following	1.69	7	1.91	7	1.91	7	1.8	7	1.6	7	1.77	7	1.13	7

### Factors Involved in Developing Connection (Intimacy and Rapport)

**Figure 3.**
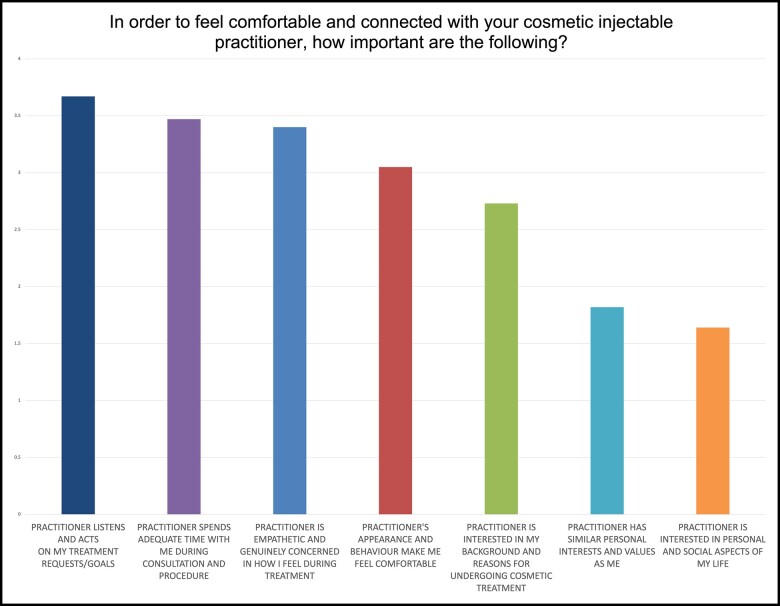
Chart showing relative importance of factors in developing connection with cosmetic injectable practitioner.

The respondents rated the importance of 6 factors for feeling comfortable and connected with their practitioner. The percentage of respondents that selected each level of importance is shown in Table 2. The relative weighting of each factor, shown in Figure 3, is calculated as an average score where not important is assigned a score of 1, up to very important, at a score of 4. 981 participants completed this question.

**Table 2.** Relative Importance to Respondents (%) for Each Factor in Feeling Comfortable and Connected With Their Cosmetic Injectable Practitioner

Factor	Not important	Somewhat important	Quite important	Very important	Weighted average
Practitioner listens and acts on my treatment requests/goals	0.5%	4.9%	21.6%	73.0%	3.67
Practitioner spends adequate time with me during consultation and procedure	1.2%	9.3%	31.1%	58.4%	3.47
Practitioner is empathetic and genuinely concerned in how I feel during treatment	1.3%	10.8%	34.2%	53.7%	3.4
Practitioner's appearance and behavior make me feel comfortable	2.6%	23.6%	40.0%	33.9%	3.05
Practitioner is interested in my background and reasons for undergoing cosmetic treatment	10.9%	32.4%	29.9%	26.8%	2.73
Practitioner has similar personal interests and values as me	47.0%	30.9%	14.8%	7.3%	1.82
Practitioner is interested in personal and social aspects of my life	54.5%	30.7%	10.6%	4.2%	1.64

## DISCUSSION

### Factors in Development of Trust

The CIPEES survey found the standout factor for the development of trust in a practitioner to be *qualifications and training,* with 46% of respondents rating this as the most important factor and a weighted score of 5.71. This corresponds with credibility in the trustworthiness equation. The lowest ranked factor was practitioner's active online presence and social media following with a weighted score 1.79. An online presence can represent a manifestation of self-orientation, in which case it becomes a negative factor in the accrual of trustworthiness.^10^ Online presence, however, may not always be a negative factor in building trust and may positively contribute to a practitioner's reputation, which was considered the second most important factor in trusting a practitioner.

### Factors in the Development of Rapport

The highest rated factor in developing rapport was listening (practitioner listens and acts on my treatment requests/goals), with 73% of respondents rating it as very important and 22% as important (relative weighting of 3.67). Following this was a time (practitioner spends adequate time with me during consultation and procedure), with 58% of respondents rating it as very important and 31% as important (relative weighting 3.47). Also highly rated was the practitioner's concern for the patient during the procedure (practitioner is empathetic and genuinely concerned in how I feel during treatment), with 54% of respondents rating it as very important and 34% as quite important (relative weighting 3.40).

The least important factors were the practitioner having the same interests and values as the respondent, with only 7% of respondents rating it as very important, compared to 47% as not important (relative weighting 1.82); and finally, the practitioner's interest in personal and social aspects of the respondent's life, with 4% respondents rating this as very important compared to 55% not important (weighting 1.64). The practitioner's interest in the respondent's background and reasons for undergoing cosmetic treatment, and the respondent feeling comfortable with practitioner's appearance and behavior were rated moderately (relative weightings of 2.73 and 3.05, respectively).

These findings suggest that giving adequate time listening and empathy are the most important factors in developing rapport with the cosmetic injectable patient. The authors do however acknowledge the limitations of this study, particularly with regard to the inherent selection and response bias with an online survey format, and the vastly heterogeneous group of participants. Additionally, the majority of respondents were females living in either Australia, the United Kingdom, or the United States. It is possible that the factors in building trust and trustworthiness could differ between gender and also cultures. Ideally, prospective qualitative research and case-controlled studies would more accurately elucidate whether there are differences in needs of different patient groups.

### Trustworthiness Equation

The trustworthiness equation may be used as an assessment framework for improving the ability to build trustworthiness in interpersonal relationships. Although originally targeting business professionals in advisory and consulting roles, it is highly relevant to aesthetic clinicians and healthcare professionals (HCPs). The novice HCP may balk at the notion of having their proficiency based on anything other than technical competence, as the latter often represents the initial primary focus. However, transitioning to full proficiency mandates automatic integration of initial technical aspects and sequential accrual of a finessed spectrum of technical skills, observational powers, and relational mindset.

The trustworthiness equation uses 4 objective variables to measure trustworthiness, equating trustworthiness to the sum of credibility, reliability, and intimacy, divided by the level of self-orientation. Although technical brilliance may be sufficient to induce high levels of patient trust in pure medicine, business and service-oriented fields such as aesthetic medicine require proficiency across all 4 areas, in addition to awareness of weaknesses.

Credibility, often the primary focus of medical professionals, is rational, easily understood and increases with qualifications, competency, credentials, reputation, recommendation, expertise, and seniority. In medicine, credibility tends to be naturally high and develops over time with continuing professional development yet is not amenable to quick fixes, requiring consistent self-evaluation and betterment. Authentic credentialization of expert status and personal recommendation are additional factors. Tellingly, for maximum credibility from a patient perspective, this expertise needs to be accompanied by effective communication and the ability to convey knowledge.

Reliability is defined by actions, dependability, and behavioral consistency. It may require multiple touchpoints for accurate assessment, thus being informed through previous interactions. Reliability links intention to action, words, and deeds. The term implies being capable of delivering on a promise, maintaining confidence, and respecting others. Opinions regarding reliability unconsciously develop through the predictability of habits, preferences, or expectations.

Service provider reliability constitutes a conscious choice. Critically, because operating beyond the circle of competence inevitably erodes reliability, reliability inherently mandates the ability to say no. Conversely, because setting realistic expectations frequently portends the ability to fulfill them, this forms the essence of reliability. People-pleasing and the inability to set realistic expectations are common reliability traps. Both over-promising and glossing over potential complications are best avoided, while understanding and relating to patient goals and preferences are encouraged due to fostering reliability.

In contrast to reliability and credibility, intimacy and self-orientation are conceptually complex and often less readily achievable. Intimacy, also known as rapport, is the most common deficit when failing to achieve the trust necessary to communicate around difficult topics. Sadly, HPCs often pride themselves on being emotionally unattached, thus emanating aloofness.^12^

**Figure 4.**
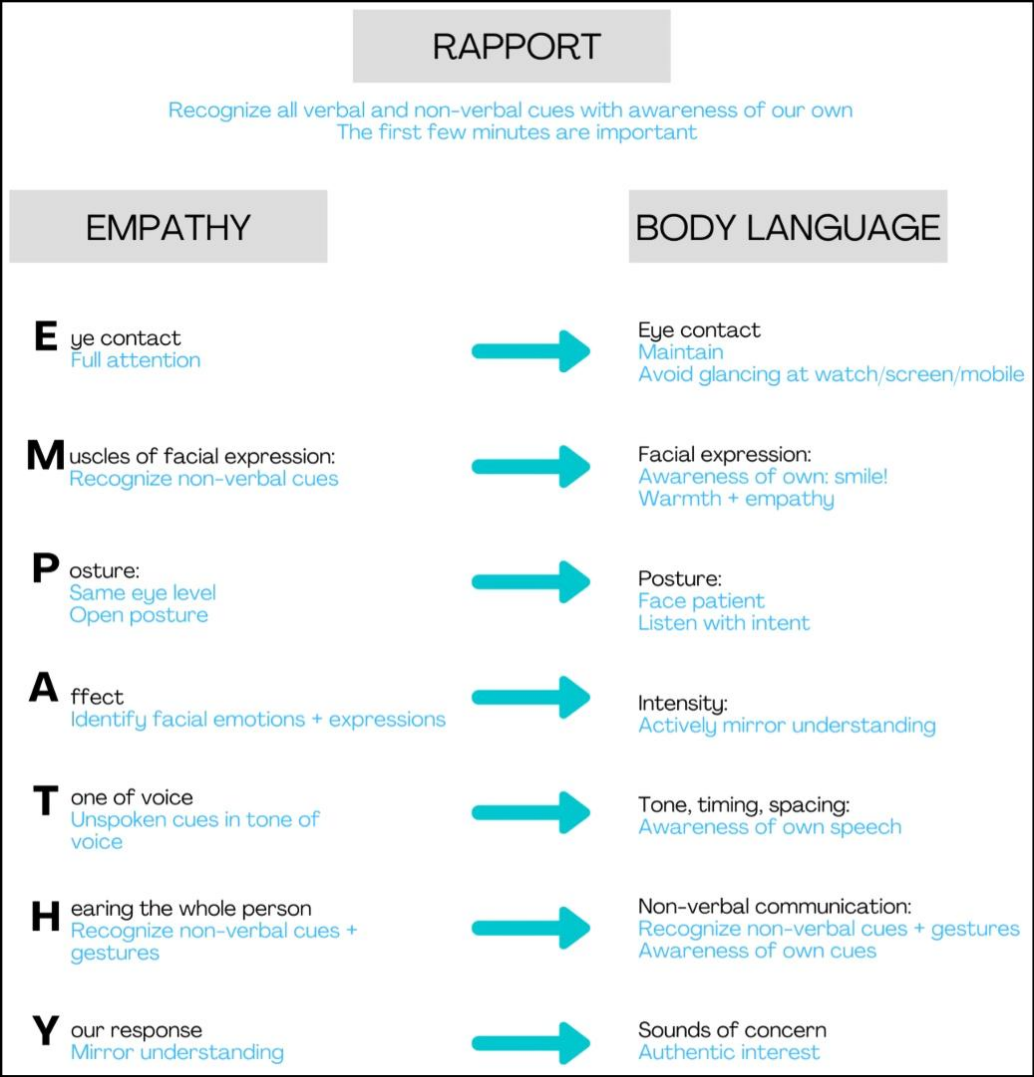
Illustration of the correlation between empathy and rapport (positive body language).

Rapport, or the measure of interpersonal comfort and connectedness during communication, is difficult to define and measure. Yet, it is considered the most powerful component of trustworthiness. Rapport is accrued through a series of verbal and nonverbal means which, interestingly, correlate closely to the factors portraying empathy (Figure 4).

Effective rapport encourages patients to freely share personal and private information, emotions, fears, insecurities, ambitions, and desires. Empathic listening effectively mitigates the fear of showing vulnerability. Empathy and rapport are built through feeling both heard and understood builds. Sadly, this is a frequent failing when professionals allow insufficient consultation time, attempt to prove their expertise, or fail to truly listen to understand. This frequently translates as pompous, assertive, insensitive, or unperceptive behavior which actively erodes trust.

Rapport correlates closely with empathy, has a bearing on the specific issues at hand, is motivated by emotional honesty, and by no means implies crossing professional boundaries. Perceptive observation of clues pertaining to the patient's appearance, objectives, goals, motivations, self-esteem, and confidence are sought. Because it implies vulnerability, rapport may be daunting to both parties, requiring adequate emotional intelligence from the side of the provider.

Although optimal uptake of advice is facilitated by empathy and rapport, the dominance of professional content mastery at the expense of empathy and active listening remains the greatest challenge in patient communication. Creating rapport requires courage, practice, and authentic interest in the patient and, despite the challenges, constitutes one of the quickest routes to achieving trustworthiness. There is a convincing body of evidence supporting the fact that empathy can and should be trained and that this has long-lasting clinical implications for patient wellness and satisfaction.^13–15^

Self-orientation (self-interest), the denominator, unfortunately, constitutes a ubiquitous negative trait among aesthetic practitioners. It is commonly signaled by factors such as preoccupation with own image and agenda, excessive online presence, and focus on financial reward. Typical features of self-orientation cited to actively erode trust are presented in Table 3.^10^ Constant, conscious, and honest introspection is mandatory for control of this surreptitious trait. Conversely, low levels of self-orientation are demonstrated by allowing the patient to speak, listening with full attention, reflective and empathic listening, using open-ended questions, defining the problem rather than launching directly into a solution, taking responsibility for failed communications, and admitting what you do not know or cannot do.

**Table 3.** Features Recognized as Excessive Self-Orientation

Features
A tendency to relate stories to ourselves
An unwillingness to say we do not know
A need to appear on top of things
A desire to look at intelligent, clever, or witty
Name dropping
A desire to jump to the solution or give answers too quickly
A desire to win that exceeds the desire to help the client
A recitation of qualifications or status
A desire to be right
Passive listening
Fears of various kinds: not knowing; not having the right answer; not appearing intelligent; being rejected

The consultation process arguably constitutes the most vital time for building an empathic connection. With the relationship between patient and HCP increasingly deemed a partnership involving shared decision-making, good communication skills are paramount in improving mutual understanding, patient satisfaction, and subsequent retention.^16^

**Figure 5.**
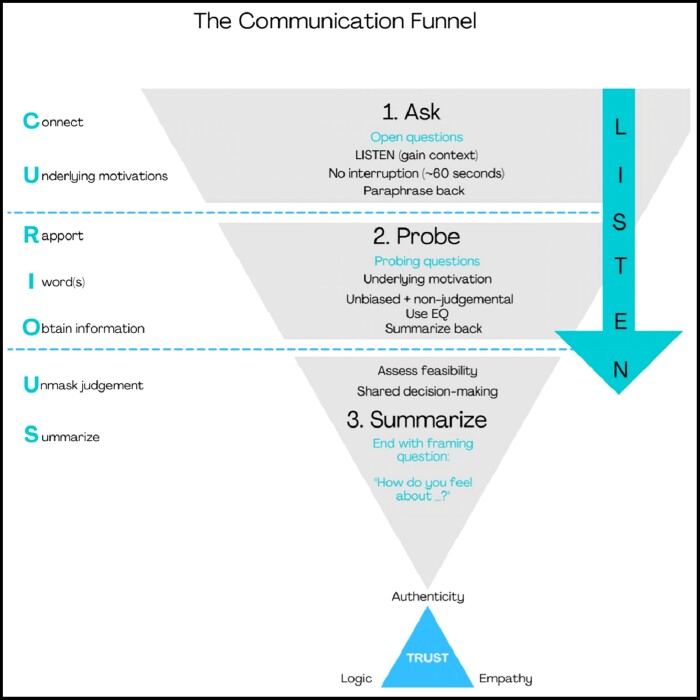
Stages of the empathic communication funnel. Note the question types best suited to each of the 3 stages.

Undoubtedly, skilled aesthetic consultation has become a discipline of its own. The factors positively impacting empathic connection may be practically implemented by following the stages of the Empathic Communication Funnel (Figure 5). The initial stage involves building connections and elucidating optimal information by asking open questions. The second stage encourages teasing out underlying motivations through probing, reflective, and hypothetical questions. During the third stage, shared decision-making is finessed. The inherent components are summarized by the acronym CURIOUS (Figure 5).

Connection is built through listening with authentic curiosity to truly understand. Underlying motivations are sought through asking the correct questions and observing unspoken behaviors. This is particularly important in recognizing underlying body dysmorphic disorder (BDD). Rapport may be built through projecting both nonverbal and verbal cues and correlating closely with an expression of empathy. The I-word, signaling the practitioner's own opinion, should preferably not be used during the first 2 stages as it is prone to blocking the flow of volunteered information. Obtaining complete information requires sufficient time allocation to the consultation process and listening with curiosity. Unmasking one's own judgment is mandatory, especially when dealing with nonstereotypic patients such as gender-diverse individuals, BDD, and generation-specific needs and requires adequate emotional intelligence. Tellingly, emotional intelligence is increasingly being cited in selection criteria for medical schools and subsequent training curricula. Other than being a vital component of resilience, effective practice, and relationships, emotional intelligence enables the HCP to accurately recognize shifts in the patient's mood and demeanor.^17^

Summarizing back at various points during the consultation, and especially at the end, ensures that the transfer of knowledge has been adequate. The realization that someone has listened attentively, and is able to feedback accurately, is a valuable pillar for building trust.

Undoubtedly, the essence of successful and empathic communication lies in listening with curiosity, truly understanding, without judgment, and with the intent to help. Importantly, trust is actively built during the third stage of this funnel while summarizing back to the patient what has been heard. Completing the consultation with the framing question, that is, one beginning with “how do you feel about… (the plan upon which we have decided during joint decision making) … further accrues trust.”

## CONCLUSIONS

In aesthetic medicine, both patient and practitioner satisfaction correlate with levels of interpersonal trust. Importantly, effective engagement implies both trust and trustworthiness.^17^ The CIPEES survey results support patient trust being built through credibility, reliability, and intimacy, with low levels of practitioner self-orientation. The *trustworthiness equation* provides a framework for identifying practitioner weaknesses in patient relationships and actionable methods of improving trustworthiness. Rapport, while being the most elusive trust factor, may be increased through the simple steps of allocating additional consultation time, and practicing authentic, focused empathic listening as described in the empathic communication funnel.

## Supplementary Material

ojac082_Supplementary_Data
