# Supplementary material for: Factors Influencing Trust and Trustworthiness: Cosmetic Injectable Patient Experience Exploratory Study (CIPEES)—Part 3
Source: Aesthet Surg J Open Forum. 2022 Nov 7;4:ojac082. doi: 10.1093/asjof/ojac082 (PMC9687811; doi:10.1093/asjof/ojac082)
Supplement: ojac082_Supplementary_Data [file ojac082_supplementary_data.zip › 22-0106_Appendix 2.docx]

**Appendix 2.**

| Cosmetic Injectables - Patient Experience |  |  |
| --- | --- | --- |
| In what country do you live? | | |
| Answer Choices | Responses | |
| Afghanistan | 0.07% | 1 |
| Albania | 0.07% | 1 |
| Algeria | 0.00% | 0 |
| Andorra | 0.00% | 0 |
| Angola | 0.07% | 1 |
| Anguilla | 0.00% | 0 |
| Antigua and Barbuda | 0.00% | 0 |
| Argentina | 0.07% | 1 |
| Armenia | 0.00% | 0 |
| Australia | 59.05% | 845 |
| Austria | 0.21% | 3 |
| Azerbaijan | 0.07% | 1 |
| Bahamas | 0.00% | 0 |
| Bahrain | 0.07% | 1 |
| Bangladesh | 0.07% | 1 |
| Barbados | 0.00% | 0 |
| Belarus | 0.00% | 0 |
| Belgium | 0.14% | 2 |
| Belize | 0.00% | 0 |
| Benin | 0.00% | 0 |
| Bhutan | 0.00% | 0 |
| Bolivia (Plurinational State of) | 0.00% | 0 |
| Bosnia and Herzegovina | 0.00% | 0 |
| Botswana | 0.00% | 0 |
| Brazil | 0.42% | 6 |
| British Virgin Island | 0.00% | 0 |
| Brunei Darussalam | 0.00% | 0 |
| Bulgaria | 0.00% | 0 |
| Burkina Faso | 0.00% | 0 |
| Burundi | 0.00% | 0 |
| Cabo Verde | 0.00% | 0 |
| Cambodia | 0.00% | 0 |
| Cameroon | 0.07% | 1 |
| Canada | 3.00% | 43 |
| Cayman Islands | 0.00% | 0 |
| Central African Republic | 0.00% | 0 |
| Chad | 0.00% | 0 |
| Chile | 0.00% | 0 |
| China | 0.00% | 0 |
| Colombia | 0.14% | 2 |
| Comoros | 0.00% | 0 |
| Congo | 0.00% | 0 |
| Costa Rica | 0.07% | 1 |
| Côte D'Ivoire | 0.00% | 0 |
| Croatia | 0.00% | 0 |
| Cuba | 0.00% | 0 |
| Cyprus | 0.07% | 1 |
| Czech Republic | 0.07% | 1 |
| Democratic People's Republic of Korea | 0.00% | 0 |
| Democratic Republic of the Congo | 0.00% | 0 |
| Denmark | 0.28% | 4 |
| Djibouti | 0.00% | 0 |
| Dominica | 0.00% | 0 |
| Dominican Republic | 0.07% | 1 |
| Ecuador | 0.00% | 0 |
| Egypt | 0.07% | 1 |
| El Salvador | 0.00% | 0 |
| Equatorial Guinea | 0.00% | 0 |
| Eritrea | 0.00% | 0 |
| Estonia | 0.07% | 1 |
| Ethiopia | 0.00% | 0 |
| Fiji | 0.00% | 0 |
| Finland | 0.00% | 0 |
| France | 0.28% | 4 |
| Gabon | 0.00% | 0 |
| Gambia | 0.00% | 0 |
| Georgia | 0.07% | 1 |
| Germany | 1.05% | 15 |
| Ghana | 0.00% | 0 |
| Greece | 0.35% | 5 |
| Grenada | 0.00% | 0 |
| Guatemala | 0.00% | 0 |
| Guinea | 0.00% | 0 |
| Guinea Bissau | 0.00% | 0 |
| Guyana | 0.00% | 0 |
| Haiti | 0.00% | 0 |
| Holy See | 0.00% | 0 |
| Honduras | 0.07% | 1 |
| Hungary | 0.00% | 0 |
| Iceland | 0.07% | 1 |
| India | 1.61% | 23 |
| Indonesia | 0.77% | 11 |
| Iran (Islamic Republic of) | 0.00% | 0 |
| Iraq | 0.21% | 3 |
| Ireland | 0.70% | 10 |
| Israel | 0.14% | 2 |
| Italy | 0.35% | 5 |
| Jamaica | 0.00% | 0 |
| Japan | 0.14% | 2 |
| Jordan | 0.00% | 0 |
| Kazakhstan | 0.07% | 1 |
| Kenya | 0.00% | 0 |
| Kiribati | 0.00% | 0 |
| Kuwait | 0.07% | 1 |
| Kyrgyzstan | 0.00% | 0 |
| Lao People’s Democratic Republic | 0.00% | 0 |
| Latvia | 0.00% | 0 |
| Lebanon | 0.07% | 1 |
| Lesotho | 0.00% | 0 |
| Liberia | 0.00% | 0 |
| Libya | 0.00% | 0 |
| Liechtenstein | 0.00% | 0 |
| Lithuania | 0.00% | 0 |
| Luxembourg | 0.07% | 1 |
| Madagascar | 0.00% | 0 |
| Malawi | 0.00% | 0 |
| Malaysia | 0.63% | 9 |
| Maldives | 0.00% | 0 |
| Mali | 0.00% | 0 |
| Malta | 0.00% | 0 |
| Marshall Islands | 0.00% | 0 |
| Mauritania | 0.00% | 0 |
| Mauritius | 0.07% | 1 |
| Mexico | 0.28% | 4 |
| Micronesia (Federated States of) | 0.00% | 0 |
| Monaco | 0.00% | 0 |
| Mongolia | 0.00% | 0 |
| Montenegro | 0.00% | 0 |
| Montserrat | 0.00% | 0 |
| Morocco | 0.07% | 1 |
| Mozambique | 0.00% | 0 |
| Myanmar | 0.07% | 1 |
| Namibia | 0.00% | 0 |
| Nauru | 0.00% | 0 |
| Nepal | 0.07% | 1 |
| Netherlands | 0.56% | 8 |
| New Zealand | 2.31% | 33 |
| Nicaragua | 0.00% | 0 |
| Niger | 0.00% | 0 |
| Nigeria | 0.00% | 0 |
| Norway | 0.35% | 5 |
| Oman | 0.00% | 0 |
| Pakistan | 0.28% | 4 |
| Palau | 0.00% | 0 |
| Panama | 0.14% | 2 |
| Papua New Guinea | 0.00% | 0 |
| Paraguay | 0.07% | 1 |
| Peru | 0.00% | 0 |
| Philippines | 0.35% | 5 |
| Poland | 0.42% | 6 |
| Portugal | 0.00% | 0 |
| Qatar | 0.14% | 2 |
| Republic of Korea | 0.07% | 1 |
| Republic of Moldova | 0.00% | 0 |
| Romania | 0.84% | 12 |
| Russian Federation | 0.21% | 3 |
| Rwanda | 0.00% | 0 |
| Saint Kitts and Nevis | 0.00% | 0 |
| Saint Lucia | 0.00% | 0 |
| Saint Vincent and the Grenadines | 0.00% | 0 |
| Samoa | 0.00% | 0 |
| San Marino | 0.00% | 0 |
| Sao Tome and Principe | 0.00% | 0 |
| Saudi Arabia | 0.14% | 2 |
| Senegal | 0.00% | 0 |
| Serbia | 0.21% | 3 |
| Seychelles | 0.00% | 0 |
| Sierra Leone | 0.00% | 0 |
| Singapore | 0.77% | 11 |
| Slovakia | 0.21% | 3 |
| Slovenia | 0.14% | 2 |
| Solomon Islands | 0.00% | 0 |
| Somalia | 0.00% | 0 |
| South Africa | 1.96% | 28 |
| South ‎Sudan | 0.00% | 0 |
| Spain | 0.56% | 8 |
| Sri Lanka | 0.00% | 0 |
| State of Palestine | 0.00% | 0 |
| Sudan | 0.00% | 0 |
| Suriname | 0.07% | 1 |
| Swaziland | 0.00% | 0 |
| Sweden | 0.28% | 4 |
| Switzerland | 0.49% | 7 |
| Syrian Arab Republic | 0.00% | 0 |
| Tajikistan | 0.00% | 0 |
| Thailand | 0.21% | 3 |
| The former Yugoslav Republic of Macedonia | 0.00% | 0 |
| Timor-Leste | 0.00% | 0 |
| Togo | 0.00% | 0 |
| Tonga | 0.00% | 0 |
| Trinidad and Tobago | 0.07% | 1 |
| Tunisia | 0.00% | 0 |
| Turkey | 0.35% | 5 |
| Turkmenistan | 0.00% | 0 |
| Turks and Caicos | 0.00% | 0 |
| Tuvalu | 0.00% | 0 |
| Uganda | 0.00% | 0 |
| Ukraine | 0.07% | 1 |
| United Arab Emirates | 0.84% | 12 |
| United Kingdom of Great Britain and Northern Ireland | 6.22% | 89 |
| United Republic of Tanzania | 0.00% | 0 |
| United States of America | 9.99% | 143 |
| Uruguay | 0.07% | 1 |
| Uzbekistan | 0.00% | 0 |
| Vanuatu | 0.00% | 0 |
| Venezuela (Bolivarian Republic of) | 0.00% | 0 |
| Vietnam | 0.14% | 2 |
| Yemen | 0.00% | 0 |
| Zambia | 0.00% | 0 |
| Zimbabwe | 0.00% | 0 |
|  | Answered | 1431 |
