# Supplementary material for: Factors Influencing Trust and Trustworthiness: Cosmetic Injectable Patient Experience Exploratory Study (CIPEES)—Part 3
Source: Aesthet Surg J Open Forum. 2022 Nov 7;4:ojac082. doi: 10.1093/asjof/ojac082 (PMC9687811; doi:10.1093/asjof/ojac082)
Supplement: ojac082_Supplementary_Data [file ojac082_supplementary_data.zip › Appendix 1. Blank Survey.pdf]

## SURVEY CONSENT

**The purpose of this research project is to explore patient’s opinions and experiences, specific to the area of cosmetic injectables. This is a research project being conducted by Dermatologist Dr Cara McDonald. You are invited to participate in this research project if you have ever undergone cosmetic injectable treatments such as muscle relaxant injections, dermal fillers or fat dissolving injections.**

**Your participation in this research study is voluntary. You may choose not to participate. If you decide to participate in this research survey, you may withdraw at any time. If you decide not to participate in this study or if you withdraw from participating at any time, you will not be penalised.**

**The procedure involves filling in an online survey that will take approximately 10 minutes. Your responses will be kept confidential and we do not collect any identifying information such as your name, email address or IP address. The survey questions will be about your cosmetic injectable treatments.**

**We will do our best to keep your information confidential. All data is stored in a password protected electronic format. To help protect your confidentiality, the surveys will not contain information that will personally identify you. The results of this study will be used for research and educational purposes only and will aggregated before being shared or published.**

**This research has been reviewed and approved by St Vincent’s Hospital Melbourne Human Research Ethics Committee. View the SurveyMonkey Privacy Policy.**

* 1. **ELECTRONIC CONSENT: Please select your choice below.**

Clicking on the "agree" button below indicates that:

- you have read the above information
- you voluntarily agree to participate
- you are at least 18 years of age

If you do not wish to participate in the research study, please decline participation by clicking on the "disagree" button.

- ◯ Agree
- ◯ Disagree

**Thank you for agreeing to participate in this short survey. We are interested in your personal opinions regarding cosmetic injectable treatments. This survey will form part of a research project where your responses will be aggregated with others and will remain anonymous.**

2. Please select your age range

- ◯ 18-24
- ◯ 25-34
- ◯ 35-44
- ◯ 45-54
- ◯ 55-64
- ◯ 65+

3. What is your gender?

- ◯ Female
- ◯ Male
- ◯ Other (specify)

4. In what country do you live?

5. With regards to cosmetic injectable treatments, please select to what degree the following statements describes your personal thoughts or feelings.

| | Does not describe me | Somewhat describes me | Describes me well |
|---|---|---|---|
| You feel that you have a **specific feature/area** that causes you to be self-conscious and needs/needed to be fixed. | ◯ | ◯ | ◯ |
| You feel that you have **aged prematurely** and are let down by your appearance. | ◯ | ◯ | ◯ |
| You are very proactive and want to **reduce all signs of ageing**. | ◯ | ◯ | ◯ |
| You want to slow down or reduce the signs of ageing but want to maintain a **natural look.** | ◯ | ◯ | ◯ |
| You place a high value on your appearance and are aiming for **"next level" beautification.** | ◯ | ◯ | ◯ |
| You love the **cosmetically-enhanced** and "done" look. | ◯ | ◯ | ◯ |
| You are **critical of your own appearance** and either avoid looking looking in the mirror or obsess over what is "wrong". | ◯ | ◯ | ◯ |
| You will **forego other things** in order to undergo more cosmetic treatments. | ◯ | ◯ | ◯ |
| You are happy to invest time and money in the **best quality** treatments and products. | ◯ | ◯ | ◯ |
| You **don't want seem vain** or look "done" but want to look more like “yourself” again. | ◯ | ◯ | ◯ |

| | Does not describe me | Somewhat describes me | Describes me well |
|---|---|---|---|
| You choose your cosmetic injectable treatments by the **best price/deal** available. | ◯ | ◯ | ◯ |
| You **worry about looking "done"** and don't want others to notice you have had treatment. | ◯ | ◯ | ◯ |
| Even after treatment, you **don't feel happy** with your appearance. | ◯ | ◯ | ◯ |

6. Do financial constraints limit your cosmetic injectable treatments?

- ◯ Yes
- ◯ Somewhat
- ◯ No
- ◯ Unsure

7. In total, how many different cosmetic injectable practitioners (including current practitioner) have you seen at any stage? Note: Practitioner refers to the individual who treated you as opposed to clinic

- ◯ 0
- ◯ 1
- ◯ 2
- ◯ 3
- ◯ 4
- ◯ 5+

* 8. Please estimate your total number of visits for cosmetic injectable treatments in any location during the last 2 years.

- ◯ 0 visits
- ◯ 1-3 visits
- ◯ 4-9 visits
- ◯ 10+ visits

9. Thinking about the process of selecting a cosmetic injectable practitioner, which of the following influenced your choice? (Please select all that apply)

- ☐ Social media (eg Instagram or Facebook) following or advertisement
- ☐ Searched online (google or other web browser) for clinic/practitioner or service
- ☐ Recommendation from friend, family or associate
- ☐ Advertisement in print (eg. newspaper, magazine, brochure)
- ☐ Attended clinic for other reason/s previously (eg. to see general practitioner or specialist)
- ☐ Referral from medical practitioner in another clinic (eg. specialist or general practitioner)
- ☐ Clinic convenient to me and booked directly (ie. no specific recommendation)
- ☐ Do not recall
- ☐ Have not yet chosen a practitioner
- ☐ Recommendation from industry professional (eg beauty therapist)
- ☐ Other (please specify)

10. Please indicate the qualification or profession of your most recent cosmetic injector.

- [ ] Plastic surgeon
- [ ] Cosmetic surgeon
- [ ] Cosmetic doctor
- [ ] Dermatologist
- [ ] Nurse
- [ ] Beauty Therapist
- [ ] Unsure
- [ ] General practitioner
- [ ] Dermal Clinician/Therapist
- [ ] No qualifications
- [ ] Other (please specify)

* 11. Have you attended more than one visit with your most recent practitioner for cosmetic injectables?

- ( ) Yes
- ( ) No

* 12. Thinking specifically about the reasons you have **returned** to your current cosmetic injectable practitioner, please indicate how important each of the following are to you.

| | Not important | Slightly important | Quite important | Very important |
|---|---|---|---|---|
| Convenience | ○ | ○ | ○ | ○ |
| Customer service in the clinic | ○ | ○ | ○ | ○ |
| Satisfaction with cosmetic result from previous treatment/s | ○ | ○ | ○ | ○ |
| Trust in the ability of the practitioner | ○ | ○ | ○ | ○ |
| Feel comfortable and relaxed with practitioner | ○ | ○ | ○ | ○ |

Other (please specify)

* 13. Please rate your overall **satisfaction with the cosmetic results** with your most recent injectable practitioner, where 0 is the worst cosmetic result possible and 10 is the best result possible.

| | Worst cosmetic result | 1 | 2 | 3 | 4 | 5 | 6 | 7 | 8 | 9 | Best cosmetic result |
|---|---|---|---|---|---|---|---|---|---|---|---|
| . | ◯ | ◯ | ◯ | ◯ | ◯ | ◯ | ◯ | ◯ | ◯ | ◯ | ◯ |

14. In allowing you to trust any practitioner to perform your cosmetic injectable treatments, how important are each of the following?

| | Not important | Slightly important | Quite important | Very important |
|---|---|---|---|---|
| Qualifications and training of practitioner (degree, doctor, specialist, educator, trainer, key opinion leader) | ◯ | ◯ | ◯ | ◯ |
| Personal appearance and aesthetic ideals of the practitioner | ◯ | ◯ | ◯ | ◯ |
| Reputation or recommendation of the practitioner | ◯ | ◯ | ◯ | ◯ |
| Time and education spent with me during consultation and procedure | ◯ | ◯ | ◯ | ◯ |
| Feeling comfortable and relaxed with my practitioner | ◯ | ◯ | ◯ | ◯ |
| Practitioner's active online presence and social media following | ◯ | ◯ | ◯ | ◯ |
| Seeing Before and After photos of other patients | ◯ | ◯ | ◯ | ◯ |
| Customer service and respect by the clinic staff | ◯ | ◯ | ◯ | ◯ |

Other (please specify)

[ ]

15. In order to relax and feel comfortable with your cosmetic injectable practitioner, how important are the following?

| | Not important | Somewhat important | Quite important | Very important |
|---|---|---|---|---|
| Practitioner spends adequate time during consultation and procedure | ◯ | ◯ | ◯ | ◯ |
| Practitioner engages in personal/social (non-medical) conversation | ◯ | ◯ | ◯ | ◯ |
| Practitioner listens and acts on my opinion regarding treatment options and planning | ◯ | ◯ | ◯ | ◯ |
| Practitioner is interested in my background and reasons for undergoing cosmetic treatment | ◯ | ◯ | ◯ | ◯ |
| Practitioner is empathetic and genuinely concerned in how I feel during treatment | ◯ | ◯ | ◯ | ◯ |
| Practitioner has similar interests and values as me | ◯ | ◯ | ◯ | ◯ |
| Practitioner's appearance and behaviour suit my personality | ◯ | ◯ | ◯ | ◯ |

Other (please specify)

* 16. Please rate your **level of trust** in your most recent injectable practitioner, where **trust** is your belief that they will act in your best interest. Select level from 0, which is no trust at all, to 10 which is complete trust.

| | No Trust | 1 | 2 | 3 | 4 | 5 | 6 | 7 | 8 | 9 | Complete Trust |
|---|---|---|---|---|---|---|---|---|---|---|---|
| . | ◯ | ◯ | ◯ | ◯ | ◯ | ◯ | ◯ | ◯ | ◯ | ◯ | ◯ |
